# Supplementary material for: Flavones, Flavonols, Lignans, and Caffeic Acid Derivatives from Dracocephalum moldavica and Their In Vitro Effects on Multiple Myeloma and Acute Myeloid Leukemia
Source: Int J Mol Sci. 2022 Nov 17;23(22):14219. doi: 10.3390/ijms232214219 (PMC9696366; doi:10.3390/ijms232214219)
Supplement: Supplementary file 1 [file ijms-23-14219-s001.zip › ijms-1916245-supplementary.pdf]

Supporting information

Flavones, Flavonols, Lignans, and Caffeic Acid Derivatives from *Dracocephalum moldavica* and Their In Vitro Effects on Multiple Myeloma and Acute Myeloid Leukemia

Karin Jöhrer [1], Mayra Galarza Pérez [2], Brigitte Kircher[1,3], and Serhat Sezai Çiçek [2,*]

[1] Tyrolean Cancer Research Institute, Innrain 66, 6020 Innsbruck, Austria

[2] Department of Pharmaceutical Biology, Kiel University, Gutenbergstraße 76, 24118 Kiel, Germany

[3] Department of Internal Medicine V (Hematology and Oncology), Medical University Innsbruck, Anichstraße 35, 6020 Innsbruck, Austria

* Correspondence: scicek@pharmazie.uni-kiel.de; Tel.: +49 431-880 1077

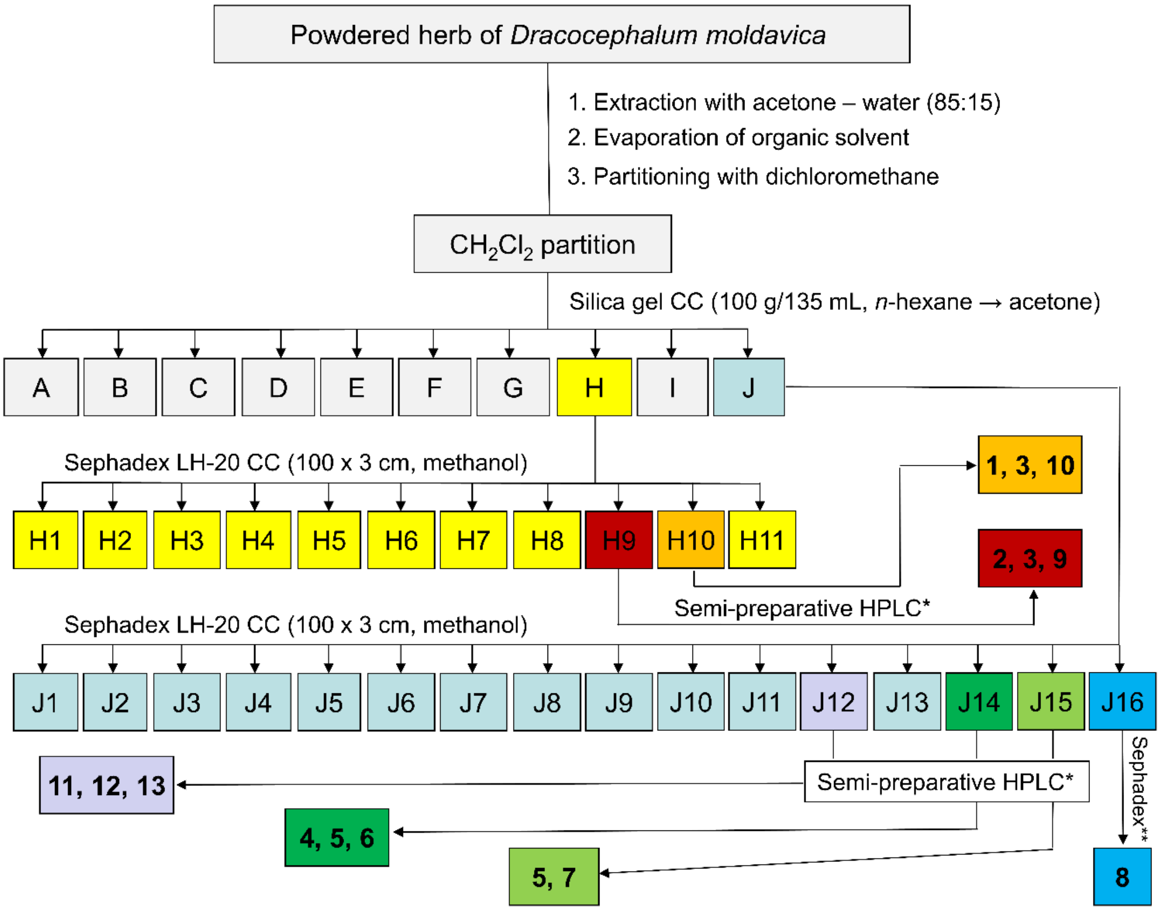

Figure S1: Isolation scheme of isolated constituents from *Dracocephalum moldavica*. *Semi-preparative HPLC was conducted on a Phenomenex Aqua column (5 µM, 250 x 10 mm) using 0.025% formic acid in water (A) and acetonitrile (B) with solvent ratios of 55:45 (fractions H9, H10, and J15), 65:35 (J14), or 70:30 (J12), respectively. **Sephadex LH-20 column chromatography of fraction J16 was conducted with methanol (100 x 1 cm column size).

NMR data, MS data, and specific rotation values of isolated constituents:

Cirsimaritin (**1**): ESIMS (positive) *m/z* = 315.1 [M + H]$^+$ (calcd for $C_{17}H_{15}O_6$, 315.1) and ESIMS (negative) *m/z* = 313.0 [M - H]$^-$ (calcd for $C_{17}H_{13}O_6$, 313.1). ^{1}H NMR (400 MHz, DMSO-d_6) δ 7.93 (d, *J* = 8.8 Hz, 2H, H-2', H-6'), 6.94 (s, 1H, H-8), 6.88 (d, *J* = 8.8 Hz, 2H, H-3', H-5'), 6.81 (s, 1H, H-3), 3.93 (s, 3H, OMe-7), 3.74 (s, 3H, OMe-6). ^{13}C NMR (100 MHz, DMSO-d_6) δ 182.60 (C-4), 164.82 (C-2), 163.79 (C-4'), 158.91 (C-7), 153.06 (C-5), 152.62 (C-9), 132.31 (C-6), 128.92 (2C, C-2', C-6'), 120.4 (C-1'), 117.04 (2C, C-3', C-5'), 105.47 (C-10), 102.40 (C-3), 92.01 (C-8), 60.45, 56.83.

5-Desmethylsinensetin (**2**): ESIMS (positive) *m/z* = 359.2 [M + H]$^+$ (calcd for $C_{19}H_{19}O_7$, 359.1). ^{1}H NMR (400 MHz, DMSO-d_6) δ 7.74 (dd, *J* = 8.8, 2.1 Hz, 1H, H-6'), 7.61 (d, *J* = 8.4 Hz, 1H, H-5'), 7.15 (d, *J* = 2.1 Hz, 1H, H-2'), 7.04 (br s, H-3), 6.99 (br s, 1H, H-8), 3.94 (s, 3H, OMe-7), 3.89 (s, 3H, OMe-3'), 3.86 (s, 3H, OMe-4'), 3.74 (s, 3H, OMe-6). ^{13}C NMR (100 MHz, DMSO-d_6) δ 182.84 (C-4), 164.01 (C-2), 159.23 (C-7), 152.91 (C-5), 152.85 (C-4'), 152.73 (C-9), 149.47 (C-3'), 132.33 (C-6), 123.41 (C-1'), 119.97 (C-6'), 111.49 (C-2'), 109.27 (C-5'), 105.78 (C-10), 103.53 (C-3), 91.40 (C-8), 59.79 (OMe-4'), 56.26 (OMe-3'), 55.71 (OMe-7), 55.54 (OMe-6).

Xanthomicrol (**3**): ESIMS (positive) *m/z* = 345.2 [M + H]$^+$ (calcd for $C_{18}H_{17}O_7$, 345.1) and ESIMS (negative) *m/z* = 343.0 [M - H]$^-$ (calcd for $C_{18}H_{15}O_7$, 343.1). ^{1}H NMR (400 MHz, DMSO-d_6) δ 7.92 (d, *J* = 9.0 Hz, 2H, H-2', 6'), 6.94 (d, *J* = 9.0 Hz, 2H, H-3', H-5'), 6.85 (s, 1H, H-3), 4.01 (s, 3H, OMe-7), 3.91 (s, 3H, OMe-8), 3.81 (s, 3H, OMe-6). ^{13}C NMR (100 MHz, DMSO-d_6) δ 182.45 (C-4), 164.44 (C-2), 162.97 (C-4'), 152.32 (C-7), 148.58 (C-5), 145.22 (C-9), 135.78 (C-6), 132.65 (C-8), 128.46 (2C, C-2', C-6'), 119.92 (C-1'), 116.45 (2C, C-3', C-5'), 106.22 (C-10), 102.21 (C-3), 61.88 (OMe-6), 61.45 (OMe-8), 60.53 (OMe-6).

Apigenin (**4**): ESIMS (positive) *m/z* = 271.1 [M + H]$^+$ (calcd for $C_{15}H_{11}O_5$, 271.1) and ESIMS (negative) *m/z* = 268.9 [M - H]$^-$ (calcd for $C_{15}H_9O_5$, 269.0). ^{1}H NMR (400 MHz, MeOH-*d*$_4$) δ 7.89 (d, *J* = 9.0 Hz, 2H, H-2', 6'), 6.87 (d, *J* = 9.0 Hz, 2H, H-3', H-5'), 6.61 (s, 1H, H-3), 6.46 (d, *J* = 2.1 Hz, 1H, H-8), 6.22 (d, *J* = 2.1 Hz, 1H, H-6). ^{13}C NMR (100 MHz, MeOH-*d*$_4$) δ 182.47 (C-4), 166.07 (C-2), 165.91 (C-7), 164.56 (C-9), 161.30 (C-4'), 158.07 (C-5), 129.48 (2C, C-2', C-6'), 122.86 (C-1'), 117.28 (2C, C-3', C-5'), 103.66 (C-10), 102.88 (C-3), 100.90 (C-6), 95.71 (C-8).

Luteolin (**5**): ESIMS (positive) *m/z* = 287.1 [M + H]$^+$ (calcd for $C_{15}H_{11}O_6$, 287.1) and ESIMS (negative) *m/z* = 284.9 [M - H]$^-$ (calcd for $C_{15}H_9O_6$, 285.0). ^{1}H NMR (400 MHz, MeOH-*d*$_4$) δ 7.43 (m, 2H, H-2', 6'), 6.96 (d, *J* = 8.8 Hz, 1H, H-5'), 6.58 (s, 1H, H-3), 6.47 (d, *J* = 1.7 Hz, 1H, H-8), 6.24 (d, *J* =1.7 Hz, 1H, H-6). ^{13}C NMR (100 MHz, MeOH-*d*$_4$) δ 182.33 (C-4), 168.80 C-7), 164.92 (C-2), 161.83 (C-5), 159.23 (C-9), 149.79 (C-4'), 145.70 (C-3'), 122.30 (C-1'), 118.79 (C-6'), 115.43 (C-5'), 112.77 (C-2'), 104.97 (C-10), 102.36 (C-3), 99.07 (C-6), 93.91 (C-8).

Diosmetin (**6**): ESIMS (positive) *m/z* = 301.1 [M + H]$^+$ (calcd for $C_{16}H_{13}O_6$, 301.1) and ESIMS (negative) *m/z* = 299.0 [M - H]$^-$ (calcd for $C_{16}H_{11}O_6$, 299.1). ^{1}H NMR (400 MHz, MeOH-*d*$_4$) δ 7.51 (d, *J* = 8.4 Hz, 1H, H-6'), 7.42 (br s, 1H, H-2'), 7.10 (d, *J* = 8.8 Hz, 1H, H-5'), 6.57 (s, 1H, H-3), 6.42 (br s, 1H, H-8), 6.20 (br s, 1H, H-6), 3.97 (OMe-4'). ^{13}C NMR (100 MHz, MeOH-*d*$_4$) δ 182.19 (C-4), 167.42 (C-2), 164.26 (C-9), 161.81 (C-7), 158.26 (C-5), 151.16 (C-4), 146.91 (C-3'), 123.82 (C-1'), 118.55 (C-6'), 112.51 (C-2'), 111.27 (C-5'), 103.21 (C-10), 102.85 (C-3), 99.67 (C-6), 94.33 (C-8), 55.10 (OMe-4').

Kaempferol (**7**): ESIMS (positive) *m/z* = 287.1 [M + H]$^+$ (calcd for $C_{15}H_{11}O_6$, 287.1) and ESIMS (negative) *m/z* = 285.0 [M - H]$^-$ (calcd for $C_{15}H_9O_6$, 285.0). ^{1}H NMR (400 MHz, MeOH-*d*$_4$) δ 8.13 (d, *J* = 8.7 Hz, 2H, H-2', 6'), 6.95 (d, *J* = 8.7 Hz, 2H, H-3', H-5'), 6.41 (d, *J* = 2.0 Hz, 1H, H-8), 6.21 (d, *J* = 2.0 Hz, 1H, H-6). ^{13}C NMR (100 MHz, MeOH-*d*$_4$) δ 175.91 (C-4), 165.80 (C-7), 161.19 (C-9), 159.31 (C-4'), 157.18 (C-5), 146.40 (C-2), 135.69 (C-3), 129.32 (2C, C-2', C-6'), 122.39 (C-1'), 114.99 (2C, C-3', C-5'), 102.79 (C-10), 98.46 (C-8), 93.51 (C-8).

Quercetin (**8**): ESIMS (positive) *m/z* = 303.0 [M + H]$^+$ (calcd for $C_{15}H_{11}O_7$, 303.0) and ESIMS (negative) *m/z* = 300.9 [M - H]$^-$ (calcd for $C_{15}H_9O_7$, 301.0). ^{1}H NMR (400 MHz, DMSO-*d*$_6$) δ 7.68 (d, *J* = 2.2 Hz, 1H, H-2'), 6.96 (dd, *J* = 8.5, 2.2 Hz, 1H, H-6'), 6.89 (d, *J* = 8.5 Hz, 1H, H-5'), 6.41 (d, *J* = 2.1 Hz, 1H, H-8), 6.19 (d, *J* = 2.1 Hz, 1H, H-6). ^{13}C NMR (100 MHz, DMSO-*d*$_6$) δ 176.39 (C-4), 164.50 (C-7), 161.19 (C-5), 156.53 (C-9), 148.13 (C-4'), 147.26 (C-2), 136.17 (C-3), 122.55 (C-1'), 120.44 (C-6'), 116.06 (C-5'), 115.52 (C-2'), 103.46 (C-10), 98.66 (C-6), 93.83 (C-8).

(+)-Piperitol (**9**): $[\alpha]^{20}_D$ +13.23 (c 0.17, MeOH). ESIMS (positive) *m/z* = 357.1 [M + H]$^+$ (calcd for $C_{21}H_{21}O_6$, 357.1). ^{1}H NMR (400 MHz, MeOH-*d*$_4$) δ 6.94 (d, *J* = 1.8 Hz, 1H, H-5), 6.88 (d, *J* = 1.8 Hz, 1H, H-2'), 6.85 (qd, *J* = 8.0, 1.7, 0.5 Hz, 1H, H-6'), 6.80 (dd, *J* = 8.1, 1.8 Hz, 1H, H-6), 6.78 (d, *J* = 8.0 Hz, 1H, H-5'), 6.77 (d, *J* = 8.2 Hz, 1H, H-5'), 5.93 (s, 2H, OMe), 4.71 (t, *J* = 4.5 Hz, 2H, H-7, H-7'), 4.23 (m, 2H, H-9b, H9'b), 3.85 (s, 3H, OMe), 3.84 (m, 2H, H-9a, H9'a), 3.12 (m, 2H, H-8, H-8'). ^{13}C NMR (100 MHz, MeOH-*d*$_4$) δ 149.41 (C-3), 149.14 (C-3'), 148.59 (C-4), 147.35 (C-4'), 136.55 (C-1'), 133.76 (C-1), 120.65 (C-6'), 119.79 (C-6), 116.09 (C-5′), 110.97 (C-2), 108.98 (C-5), 107.56 (C-2'), 102.39 (OMeO), 87.48 (C-7), 87.37 (C-7'), 72.68 (C-9), 72.60 (C-9'), 56.41 (OMe), 55.61 (C-8'), 55.35 (C-8).

(+)-9α-Hydroxysesamin (**10**): $[\alpha]^{20}_D$ +36.44 (c 0.45, $CHCl_3$). ESIMS (positive) *m/z* = 371.2 [M + H]$^+$ (calcd for $C_{20}H_{19}O_7$, 371.1). ^{1}H NMR (400 MHz, DMSO-*d*$_6$) δ 7.12 (d, *J* = 1.6 Hz, 1H, H-5), 6.93 (m, 1H, H-6), 6.92 (m, 1H, H-5'), 6.89–6.83 (m, 3H, H-2, H-2', H-6'), 6.00 (m, 4H, OMeO), 5.41 (s, 2H, H-9), 4.81 (d, *J* = 6.6 Hz, 1H, H-7'), 4.74 (d, *J* = 6.6 Hz, 1H, H-7), 4.11 (dd, *J* = 8.9, 6.1 Hz, 1H, H-9'b), 3.94 (dd, *J* = 9.0, 2.8 Hz, 1H, H-9'a), 3.00 (m, 1H, H-8'), 2.68 (br t, *J* = 7.2 Hz, 1H, H-8). ^{13}C NMR (100 MHz, DMSO-*d*$_6$) δ 147.95 (C-3), 147.76 (C-3'), 147.04 (C-4), 146.81 (C-4'), 137.85 (C-1), 136.79 (C-1'), 120.00 (C-6), 119.64 (C-6'), 108.53 (C-2), 108.20 (C-2'), 107.32 (C-5), 106.76 (C-5'), 101.39 (C-9), 101.28 (2 OMeO), 86.42 (C-7'), 83.12 (C-7), 71.88 (C-9'), 62.58 (C-8), 54.03 (C-8').

Caffeic acid (**11**): ESIMS (positive) *m/z* = 180.9 [M + H]$^+$ (calcd for $C_9H_9O_4$, 181.0) and ESIMS (negative) *m/z* = 179.1 [M - H]$^-$ (calcd for $C_9H_7O_4$, 179.0). ^{1}H NMR (400 MHz, MeOH-*d*$_4$) δ 7.41 (d, *J* = 16.7 Hz, 1H, H-7), 7.05 (d, *J* = 2.1 Hz, 1H, H-2), 6.93 (dd , *J* = 8.1, 2.0 Hz, 1H, H-6), 6.79 (d, *J* = 8.1 Hz, 1H, H-5), 6.31 (d, *J* = 16.0 Hz, 1H, H-8). ^{13}C NMR (100 MHz, MeOH-*d*$_4$) δ 173.08 (C-9), 147.05 (C-4), 145.26 (C-3), 142.07 (C-7), 127.50 (C-1), 120.68 (C-6), 119.23 (C-8), 115.02 (C-5), 113.38 (C-2).

(R)-(+)-rosmarinic acid (**12**): $[\alpha]^{20}_D$ +18.65 (c 0.23, MeOH). ESIMS (negative) *m/z* = 358.9 [M - H]$^-$ (calcd for $C_{18}H_{15}O_8$, 359.1). ^{1}H NMR (400 MHz, MeOH-*d*$_4$) δ 7.50 (d, *J* = 16.1 Hz, 1H, H-7'), 7.03 (d, *J* = 2.2 Hz, 1H, H-2'), 6.92 (dd , *J* = 8.3, 2.2 Hz, 1H, H-6'), 6.76 (d, *J* = 1.9 Hz, 1H, H-2), 6.76 (d, *J* = 8.2 Hz, 1H, H-5'), 6.65 (d, *J* = 8.0 Hz, 1H, H-5), 6.64 (dd , *J* = 8.0, 2.1 Hz, 1H, H-6), 6.27 (d, *J* = 15.9 Hz, 1H, H-8'), 5.08 (dd , *J* = 9.7, 3.2 Hz, 1H, H-8), 3.09 (dd, *J* = 3.3, 14.3 Hz, 1H, H-7b), 2.93 (dd, *J* = 9.9, 14.3 Hz, 1H, H-7a). ^{13}C NMR (100 MHz, MeOH-*d*$_4$) δ 174.51 (C-9), 169.04 (C-9'), 149.36 (C-4'),

146.78 (C-3'), 146.50 (C-7'), 145.94 (C-3), 144.79 (C-4), 131.30 (C-1), 128.05 (C-1'), 122.87 (C-6'), 121.72 (C-6), 117.47 (C-2), 116.44 (C-5'), 116.17 (C-5), 115.80 (C-8'), 115.07 (C-2'), 77.71 (C-8), 38.89 (C-7).

(R)-(+)-3´-O-methylrosmarinic acid (**13**): $[\alpha]^{20}_{D}$ +32.94 (c 0.35, MeOH). ESIMS (positive) *m/z* = 375.1 $[M + H]^+$ (calcd for $C_{19}H_{19}O_8$, 375.1) and ESIMS (negative) *m/z* = 372.9 $[M - H]^-$ (calcd for $C_{19}H_{17}O_8$, 373.1). ^{1}H NMR (400 MHz, MeOH-d_4) δ 7.56 (d, *J* = 16.2 Hz, 1H, H-7), 7.17 (d, *J* = 1.6 Hz, 1H, H-2), 7.05 (dd, *J* = 8.2, 1.9 Hz, 1H, H-6'), 6.80 (d, *J* = 8.3 Hz, 1H, H-5), 6.77 (d, *J* = 1.7 Hz, 1H, H-2'), 6.66 (m, 1H, H-5'), 6.63 (m, 1H, H-6), 6.35 (d, *J* = 15.8 Hz, 1H, H-8), 5.11 (dd , *J* = 9.7, 3.2 Hz, 1H, H-8'), 3.89 (s, 3H, OMe-3), 3.11 (m, 1H, H-7'b), 2.94 (m, 1H, H-7'a). ^{13}C NMR (100 MHz, MeOH-d_4) δ 175.81 (C-9), 167.57 (C-9'), 149.03 (C-4'), 147.95 (C-3'), 145.15 (C-7'), 144.61 (C-3), 143.47 (C-4), 129.71 (C-1), 126.62 (C-1'), 122.64 (C-6'), 120.35 (C-6), 116.13 (C-2), 115.02 (C-5'), 114.81 (C-5), 114.62 (C-8'), 110.33 (C-2'), 76.08 (C-8), 55.07 (OMe-3'), 38.40 (C-7).
